# The influence of the treatment response on the impact of resection margin status after preoperative chemoradiotherapy in locally advanced rectal cancer

**DOI:** 10.1186/1471-2407-13-576

**Published:** 2013-12-05

**Authors:** Joo Ho Lee, Eui Kyu Chie, Kyubo Kim, Seung-Yong Jeong, Kyu Joo Park, Jae-Gahb Park, Gyeong Hoon Kang, Sae-Won Han, Do-Youn Oh, Seock-Ah Im, Tae-You Kim, Yung-Jue Bang, Sung W Ha

**Affiliations:** 1 Department of Radiation Oncology, Seoul National University College of Medicine, Seoul, Korea; 2 Department of Surgery, Seoul National University College of Medicine, Seoul, Korea; 3 Department of Pathology, Seoul National University College of Medicine, Seoul, Korea; 4 Department of Internal Medicine, Seoul National University College of Medicine, Seoul, Korea; 5 Institute of Radiation Medicine, Medical Research Center, Seoul National University, Seoul, Korea

**Keywords:** Rectal cancer, Preoperative chemoradiotherapy, Resection margin, Treatment response

## Abstract

**Background:**

Circumferential resection margin (CRM) and distal resection margin (DRM) have different impact on clinical outcomes after preoperative chemoradiotherapy (CRT) followed by surgery. Effect and adequate length of resection margin as well as impact of treatment response after preoperative CRT was evaluated.

**Methods:**

Total of 403 patients with rectal cancer underwent preoperative CRT followed by total mesorectal excision between January 2004 and December 2010. After applying the criterion of margin less than 0.5 cm for CRM or less than 1 cm for DRM, 151 cases with locally advanced rectal cancer were included as a study cohort. All patients underwent conventionally fractionated radiation with radiation dose over 50 Gy and concurrent chemotherapy with 5-fluorouracil or capecitabine. Postoperative chemotherapy was administered to 142 patients (94.0%). Median follow-up duration was 43.1 months.

**Results:**

The 5-year overall survival (OS), disease-free survival (DFS), distant metastasis-free survival (DMFS) rates, and locoregional control rates (LRC) were 84.5%, 72.8%, 74.2%, and 86.3%, respectively. CRM of 1.5 mm and DRM of 7 mm were cutting points showing maximal difference in a maximally selected rank method. In univariate analysis, CRM of 1.5 mm was significantly related with worse clinical outcomes, whereas DRM of 7 mm was not. In multivariate analysis, CRM of 1.5 mm, and ypN were prognosticators for all studied endpoints. However, CRM was not a significant prognostic factor for good responders, defined as patients with near total regression or T down-staging, which was found in 16.5% and 40.5% among studied patients, respectively. In contrast, poor responders demonstrated a significant difference according to the CRM status for all studied end-points.

**Conclusions:**

Close CRM, defined as 1.5 mm, was a significant prognosticator, but the impact was only prominent for poor responders in subgroup analysis. Postoperative treatment strategy may be individualized based on this finding. However, findings from this study need to be validated with larger cohort.

## Background

Resection margin (RM) of rectal cancer is a well-known and strong prognostic factor for survival as well as recurrence [1,2]. However, recent strategies of preoperative treatment comprised of various modalities influence the significance of RM. Among them, long-course chemoradiotherapy (CRT) has different features compared to other approaches. Polish study reported that long-course CRT significantly reduced RM involvement and increased pathological complete remission rate over short-course radiation alone [3]. Thus, significance and adequate length of RM after long-course CRT should be re-evaluated in patients receiving long-course preoperative CRT.

In addition, several studies evaluated the relation with other factors and treatment approaches for patients with positive circumferential resection margin (CRM) [4,5] whereas many previous studies suggested only the prognostic effects of CRM [1,6-10]. In above mentioned studies, it was found that additional postoperative radiotherapy could not compensate the negative impact of positive CRM [4,5]. To investigate the biology of CRM and the relation with treatment approach, present study hypothesized that significance of positive RM could be determined by tumor biology of residual tumor cells. Tumor biology or responsiveness to anti-cancer therapy could be represented as degree of treatment response after preoperative CRT. Results from EORTC 22921 have shown that patients downstaged by preoperative CRT are more likely to benefit from adjuvant chemotherapy [18]. As tumor regression is one of the distinct features of long-course CRT over short-course radiotherapy or up-front surgery [3], in the setting of long-course preoperative CRT, impact of RM needs to be evaluated in relation to treatment response.

Present study was carried out to evaluate the effect and adequate length of RM in patients who underwent conventionally fractionated preoperative CRT for rectal cancer. In addition, effect of treatment response after preoperative CRT was assessed in relation to RM status.

## Methods

### Patients

After the approval of the institutional ethical review board of Seoul National University Hospital, medical records of 403 patients with rectal cancer who underwent preoperative CRT followed by total mesorectal excision between January 2004 and December 2010 were retrospectively reviewed. Inclusion criteria were: (1) histologically confirmed primary rectal cancer, (2) cT3-4 or N + without clinical evidence of distant metastasis, (3) total mesorectal excision following preoperative CRT, (4) close RM less than 0.5 cm for CRM or less than 1.0 cm for distal resection margin (DRM). There were 151 cases meeting the inclusion criteria.

Patient characteristics are shown in Table 1. In all patients, the clinical workup included digital rectal examination, complete blood count, liver function test, carcinoembryonic antigen (CEA) level, colonoscope, computed tomography (CT) of the chest and abdomino-pelvis. Magnetic resonance imaging (MRI) of the pelvis and whole body positron-emission tomography (PET) were performed in 149 patients (98.6%) and 30 patients (19.9%), respectively. Pathologic confirmation of primary lesion was done prior to preoperative CRT for all patients.

**Table 1:** Patient and treatment characteristics

**Characteristics**	**Value (%)**	
Median age, years (range)	56 (27–78)	
Gender		
Male	106 (70.2)	
Female	45 (29.8)	
ECOG		
0	40 (26.5)	
1	110 (72.8)	
2	1 (0.7)	
Clinical T stage		
T1/T2	0 (0.0)/14 (9.3)	
T3/T4	126 (83.5)/11 (7.2)	
Clinical N stage		
N (−)	31 (20.5)	
N (+)	120 (79.5)	
Distance from anal verge (cm)		
≤ 5 cm	120 (79.5)	
> 5 cm	31 (20.5)	
Pretreatment CEA		
Normal (≤ 5 ng/ml)	107 (70.9)	
Elevated (> 5 ng/ml)	44 (29.1)	
Median radiation dose, Gy (range)	50.4 (50.4–55.8)	
Combined chemotherapy		
5-fluorouracil	133 (88.1)	
Capecitabine	18 (11.9)	
Type of surgery		
Low anterior resection	139 (92.1)	
Abdominoperineal resection	12 (7.9)	
Pathology		
Adenocarcinoma	143 (94.7)	
Mucinous carcinoma	7 (4.6)	
Signet ring cell carcinoma	1 (0.7)	
ypT stage		
Tis/T1	3 (2.0)/10 (6.6)	
T2/T3	49 (32.5)/89 (58.9)	
ypN stage		
N0	94 (62.3)	
N1	47 (31.1)	
N2	10 (6.6)	
Lymphatic invasion		
Yes	19 (12.6)	
No	132 (87.4)	
Vascular invasion		
Yes	7 (4.6)	
No	144 (95.4)	
Perineural invasion		
Yes	23 (15.2)	
No	128 (84.8)	

Values in parentheses are percentages unless indicated otherwise. Gy, Gray; CEA, carcinoembryonic antigen.

### Treatment

Following the diagnosis of rectal cancer, all 151 patients underwent preoperative concurrent CRT for rectal cancer. The reasons for preoperative treatment were as follows: locally advanced tumor invasion (cT3-4) in 137 patients, clinically positive lymph node with cT2 in 14 patients.

All patients underwent CT simulation in prone treatment position. The gross tumor volume (GTV), consisting of all detectable tumors and suspicious lymph node, was determined from the endoscopy, CT, MRI, and PET finding. Initial clinical target volume (CTV) covered GTV and mesorectal tissues with craniocaudal extension and regional lymphatics including the perirectal, presacral, and the both internal iliac nodes. The initial planning target volume for large field (PTV-LF) included the initial CTV plus a 1 cm margin. Reduced CTV included primary lesion harboring mesorectal tissues with craniocaudal extension and grossly enlarged lateral pelvic lymph node. The secondary PTV for reduced field (PTV-RF) was also expanded for 1 cm from the reduced CTV. The initial radiotherapy for PTV-LF consisted of 25 fractions of 1.8 Gy (median: 45 Gy). The supplemental boost to PTV-RF consisted of 3–6 fractions of 1.8 Gy (range: 5.4–10.8 Gy), so total dose was 50.4–55.8 Gy (median: 50.4 Gy). Boost dose beyond 5.4Gy was offered to patients with initial cT4 presentation or limited mobility on physical examination midway through pre-operative treatment. All patients underwent concurrent chemotherapy with radiation, consisting of a 5-fluorouracil (*n* = 133) or capecitabine (*n* = 18). Most patients (n = 133) underwent a 5-fluorouracil 500 mg/m^2^ intravenous (IV) bolus injection for 3 days during week 1 and 5 of CRT, and 18 patients received capecitabine 1,650 mg/m^2^ daily on days with radiotherapy.

Total mesorectal excision was performed 5–12 weeks (median: 8.1 weeks) after preoperative CRT. Postoperative chemotherapy was administered to 142 patients (94.0%). The reasons for not undergoing post-operative chemotherapy were as follows: patient refusal in 2 patients, comorbidities or old age in 4 patients, wound problem in 1 patient, and transfer to other hospital in 2 patients. The regimens of postoperative chemotherapy were fluouracil-leucovorin (*n* = 111), capecitabine (*n* = 21), or FOLFOX (*n* = 10). Fluouracil-leucovorin regimen was 6 cycles of 5-fluorouracil 400 mg/m^2^ IV bolus and leucovorin 20 mg/m^2^ IV bolus for 5 days every 4 weeks. Capectabine was given 1250 mg/m^2^ twice daily without drug holiday for 2 weeks followed by one week rest repeated every 3 weeks upto 8 cycles for 6 months. FOLFOX regimen was either FOLFOX-4 or modified FOLFOX-6. Each cycle of FOLFOX-4 consisted of oxaliplatin (85 mg/m2) on day 1 and folinic acid (200 mg/m2) and a bolus of 5-FU (400 mg/m2) followed by a 22-hr infusion of 5-FU (600 mg/m2) on days 1 and 2, which was repeated every 2 weeks. Modified FOLFOX-6 consisted of oxaliplatin (85 mg/m2), folinic acid (400 mg/m2) and a bolus of 5-FU (400 mg/m2) followed by a 46-hr infusion of 5-FU (2400 mg/m2) repeated every 2 weeks.

### Pathologic evaluation

Surgical specimens were evaluated by pathologists to estimate and grade the pathologic responses of CRT. The pathologic responses were categorized into 4 tiers as reported previously [11]. No regression was defined as no evidence of radiation-related changes (fibrosis, necrosis, vascular change). Minimal regression was defined as dominant tumor mass with obvious radiation-related changes. Moderate regression was defined as dominant radiation-related changes with residual tumor. Near total regression was defined as microscopic residual tumor in fibrotic tissue. This grading system evaluates tumor regression grade on the basis of proportion between radiation change and residual tumor burden similar to that of Dworak’s system [12]. Thus, no regression, minimal regression, moderate regression, and near total regression correspond to grade 0, 1, 2, and 3 of Dworak’s system, respectively.

The CRM and DRM of the surgical specimens were inked and fixed in formalin. The resected specimens were sliced and measured by ruler. When the CRM taken from gross section is below 2 mm, microscopic measurement was performed to evaluate the exact length in a tenth of a millimeter. Sufficient blocks of the primary tumor and lymph nodes related to CRM were taken. When the tumor, lymph node, vascular invasion, or tumor satellites were found close to the margin, microscopic measurement was repeated to validate the exact length of RM.

To evaluate the relationship between the effect of CRM and treatment response to preoperative CRT, patients were arbitrarily divided into two subgroups: good responders and poor responders. Good responder was defined as patients showing near total regression or down-staging of T stage, whereas poor responder was defined as patients showing none of two features.

### Statistical analysis

Overall survival (OS) was defined as the time from the first date of treatment to the date of death from any cause, with survivors being censored at the time of last follow-up. Disease-free survival (DFS) was calculated as the interval from the first date of treatment to any recurrent disease detection or death, whichever occurred first. Locoregional control rate (LRC) was defined as the time from the first date of treatment to the date of locoregional relapse detected in pelvic cavity. Distant metastasis-free survival (DMFS) was calculated as the interval from the first date of treatment to distant metastasis detection or death, whichever occurred first. Patients who were alive and disease free at the time of last follow-up were censored.

Survival curves were generated by the Kaplan-Meier method, and a univariate survival comparison was performed using the log-rank test. Multivariate analyses were conducted using the Cox proportional hazards model backward stepwise selection procedure. Chi-square test was used for comparison of parameters between subgroups in good responders. P-value < 0.05 was considered statistically significant. Maxstat, the maximally selected rank method in R 2.15.1 (R Development Core Team, Vienna, Austria, http://www.R-project.org) was used to identify optimal cutting points for RM [13]. Cutting points for RM as studied for all studied endpoints including, OS, DFS, LCR, and DMFS. The maximally selected rank method analyzed RM as a continuous variable.

## Results

### Treatment response and survival

As for the pathologic response to preoperative CRT, near total regression was found in 16.5% and down-staging of T stage occurred in 40.4% patients. Down-staging from cT2 to ypTis was found in 1 patient (0.7%), from cT3 to ypTis, ypT1 and ypT2 in 2 (1.3%), 10 (6.6%) and 37 patients (24.5%), and from cT4 to ypT2, and ypT3 in 2 (1.3%), and 9 patients (6.0%), respectively.

**Figure 1:**
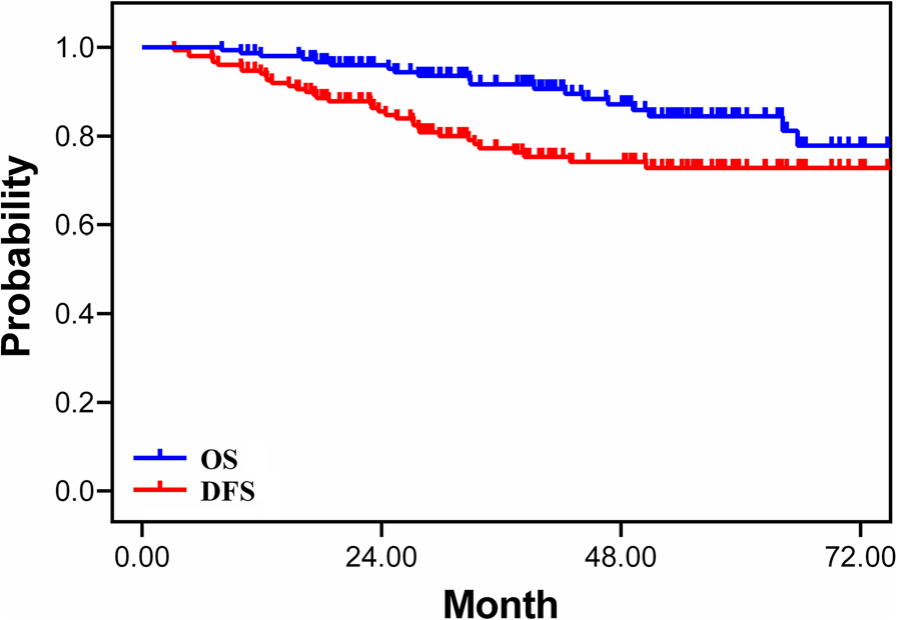
**Survival curve of all patients.** OS: overall survival, DFS: disease-free survival.

The median follow-up time for surviving patients was 43.1 months. Five-year OS, DFS, LRC, and DMFS were 84.5%, 72.8%, 86.3%, and 74.2%, respectively (Figure 1).

### The optimal cutting point and prognostic impact of resection margin

To determine which level of RM segregated patients with maximal difference of survival, a maximally selected rank method was adapted. This method found that 1.5 mm of CRM and 7 mm of DRM was the optimal cutting point for all studied end-points including OS, DFS, LRC, and DMFS. After applying the criterion of positive margin as CRM ≤1.5 mm and DRM ≤7 mm, the number of patients with positive CRM and DRM were 32 and 80, respectively. In univariate analysis, CRM of 1.5 mm was found to be a significant prognostic factor for OS (p < .001), DFS (p < .001), LRC (p < .001) and DMFS (p < .001), whereas the DRM shorter than 7 mm was not a significant prognostic factor. The results of univariate analysis are shown in Table 2.

**Table 2:** Results of univariate analysis

	**5y OS**	**p†**	**5y DFS**	**p†**	**5y LRC**	**p†**	**5y DMFS**	**p†**
Age (years)								
<60	85.7	.647	69.3	.240	82.7	.201	71.3	.349
≥ 60	82.8		80.4		94.4		80.3	
Gender								
Male	83.2	.477	70.4	.412	84.0	.365	70.4	.155
Female	87.7		79.2		92.5		84.1	
ECOG score								
0	87.5	.561	80.0	.388	94.9	.218	80.0	.506
1–2	83.5		70.5		83.4		72.3	
Clinical T stage								
T2	77.9	.881	64.9	.951	90.9	.991	64.9	.705
T3	84.9		73.0		85.8		73.9	
T4	90.9		81.8		90.0		90.9	
Clinical N stage								
N (−)	90.5	.208	81.0	.229	94.7	.115	81.0	.301
N (+)	82.9		70.5		84.0		72.2	
Distance from anal verge								
≤ 2 cm	76.7	.296	67.9	.780	87.9	.199	67.9	.650
2–5 cm	83.9		70.9		76.7		72.7	
> 5 cm	92.2		78.6		92.6		80.5	
Pretreatment CEA								
≤ 5 ng/ml	86.8	.617	77.6	.162	91.0	.081	78.5	.213
> 5 ng/ml	79.9		62.8		76.6		65.3	
Type of surgery								
LAR	89.5	<.001	75.6	<.001	88.8	<.001	77.1	<.001
APR	34.3		40.0		57.1		40.0	
Patholgic response								
None/Minimal	83.7	.228	62.8	.096	78.2	.073	64.9	.125
Moderate	80.1		74.9		89.2		75.7	
Near total	95.8		86.2		93.8		86.4	
ypT stage								
Tis/T1	92.3	.080	84.6	.036	92.3	.073	84.6	.069
T2	93.3		83.8		95.6		83.8	
T3	74.8		63.1		76.9		65.4	
ypN stage								
N0	87.4	<.001	85.5	<.001	93.4	<.001	86.7	<.001
N1	85.9		53.2		76.3		55.0	
N2	57.1		36.0		57.1		36.0	
Downstage								
Yes	87.2	.120	85.1	<.001	84.3	.213	86.5	<.001
No	80.6		56.6		87.9		57.8	
Lymphatic invasion								
Yes	75.8	.065	45.1	.004	54.7	<.001	45.1	.002
No	85.6		76.9		91.4		78.5	
Vascular invasion								
Yes	71.4	.047	38.1	.026	57.1	.002	38.1	.017
No	85.1		74.7		87.7		76.2	
Perinerual invasion								
Yes	61.0	<.001	32.1	<.001	53.3	<.001	32.1	<.001
No	88.6		79.5		91.4		81.1	
Circumferential resection margin								
≤ 1.5 mm	59.4	<.001	48.4	<.001	70.7	<.001	48.4	<.001
> 1.5 mm	91.6		79.3		90.6		81.1	
Distal resection margin								
≤ 7 mm	92.1	.010	73.0	.525	88.3	.116	74.1	.559
> 7 mm	75.5		73.4		84.3		75.1	

values are percentages of patients; †log rank test; 5y, 5-year; OS, overall survival; DFS, disease-free survival; LRC, locoregional control rates; DMFS, distant metastasis-free survival; ECOG, Eastern Cooperative Oncology Group; CEA, carcinoembryonic antigen; LAR, Low anterior resection; APR,Abdominoperineal resection.

### Analysis of prognostic factors

The univariate analysis of other prognostic factors is also shown in Table 2. Type of surgery, ypN, vascular, and perineural invasion were significant prognostic factors correlated with OS (p = <0.001, <.001, 0.047, and <0.001, respectively). Type of surgery, ypT, ypN, downstage, lymphatic, vascular, and perineural invasion were significant prognostic factors for DFS (p = <0.001, 0.036, <0.001, <0.001, 0.004, 0.026, and < 0.001, respectively). Likewise, type of surgery, ypN, lymphatic, vascular, and peri-neural invasion also had significant prognostic effect on LRC and DMFS. In contrast, age, sex, performance score, clinical stage, CEA, distance of tumor from anal verge, pathologic type, and pathologic response lacked statistical significance on above mentioned various clinical end-points.

In the multivariate analysis, ypN and CRM of 1.5 mm were independent prognostic factors for prediction of OS, DFS, LRC, and DMFS. For DFS and DMFS, ypT and lymphatic invasion were statistically significant. In addition, perineural invasion was an independently significant prognostic factor for OS and LRC. In contrast to CRM, DRM of 7 mm was not significant in multivariate analysis as well as univariate analysis (Tables 2 and 3).

**Table 3:** Results of multivariate analysis

		**p**	**RR**	**95% CI**
**OS**	Type of surgery	NS		
	ypN	.016	2.24	1.16–4.34
	Vascular invasion	NS		
	Perineural invasion	.024	2.99	1.15–7.77
	CRM of 1.5 mm	.001	4.98	1.92–12.91
	DRM of 7 mm	NS		
**DFS**	Type of surgery	NS		
	ypT	.005	2.65	1.35–5.19
	ypN	<.001	2.96	1.74–5.02
	Downstaging	NS		
	Lymphatic invasion	.011	2.97	1.29–6.85
	Vascular invasion	NS		
	Perineural invasion	NS		
	CRM of 1.5 mm	.013	2.58	1.22–5.41
**LRC**	Type of surgery	NS		
	ypN	.006	2.74	1.34–5.64
	Lymphatic invasion	NS		
	Vascular invasion	NS		
	Perineural invasion	.004	4.65	1.64–13.19
	CRM of 1.5 mm	.025	3.21	1.16–8.91
**DMFS**	Type of surgery	NS		
	ypT	.007	2.55	1.29–5.06
	ypN	<.001	2.55	1.29–5.06
	Downstaging	NS		
	Lymphatic invasion	.007	3.24	1.38–7.59
	Vascular invasion	NS		
	Perineural invasion	NS		
	CRM of 1.5 mm	.009	2.78	1.29–5.97

OS, overall survival; DFS, disease–free survival; LRC, locoregional control rates; DMFS, distant metastasis–free survival; CRM, circumferential resection margin; DRM, distal resection margin.

### Different prognostic effect of CRM according to preoperative CRT response

**Figure 2:**
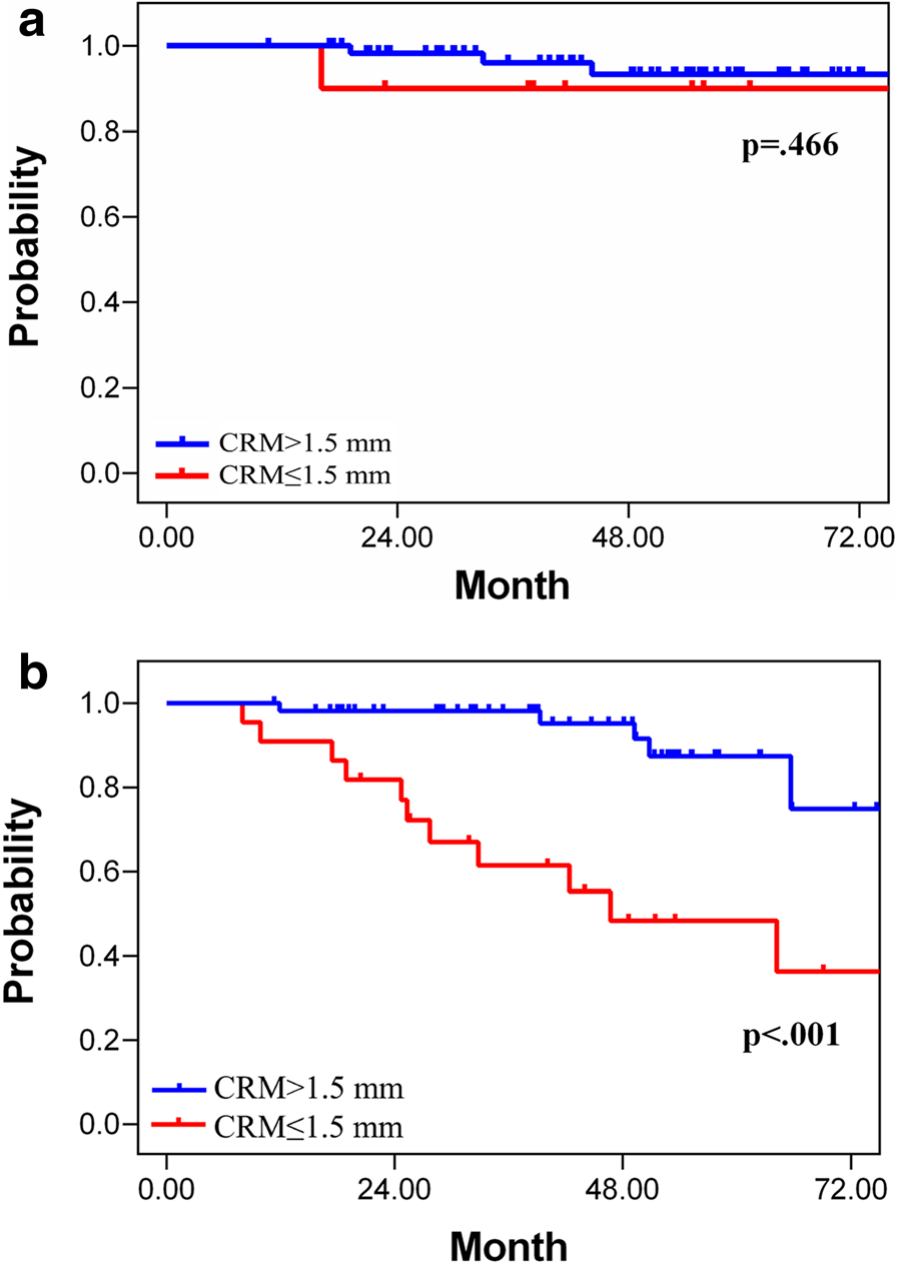
**Overall survival curve according to CRM status in good responders (a) and poor responders (b).** CRM: circumferential resection margin.

In the subgroup of good responders, CRM of 1.5 mm did not have any prognostic effect on all studied end-points. In contrast, the poor responders demonstrated a significant difference in the clinical results according to the CRM status (Table 4 and Figure 2).

**Table 4:** Subgroup analysis according to preoperative treatment response

	**Good responders**		
	**CRM > 1.5 mm**	**CRM ≤ 1.5 mm**	**p†**
Number of patients	63	10	
5-year OS	93.3	90.0	.466
5-year DFS	87.6	90.0	.948
5-year LRC	94.9	100.0	.591
5-year DMFS	89.2	90.0	.817
	**Poor responders**		
	**CRM > 1.5 mm**	**CRM ≤ 1.5 mm**	**p†**
Number of patients	56	22	
5-year OS	87.4	48.4	<.001
5-year DFS	76.0	30.5	<.001
5-year LRC	85.7	58.0	<.001
5-year DMFS	78.2	33.0	<.001

values are percentages unless indicated otherwise of patients; †log rank test. OS, overall survival; DFS, disease-free survival; LRC, locoregional control rates; DMFS, distant metastasis-free survival; CRM, circumferential resection margin.

The distribution of significant factors in multivariate analysis were compared between patients with CRM ≤1.5 mm and CRM >1.5 mm in good responders. The distribution of ypT, ypN, lymphatic invasion and perineural invasion, which were found to be significant factors in multivariate analysis, was not statistically different according to CRM status.

## Discussion

The adequate cut off point of CRM is a subject of controversy. Since the initial proposal by Quirke et al., which favored distance of 1 mm over 0 mm as cut off point [8], several large prospective studies and guidelines have adopted criteria of ≤1 mm as CRM involvement [5,14,15]. On the contrary, Natagaal et al. reported that CRM of ≤2 mm was associated with high risk for local recurrence in the series of 656 rectal cancer patients without preoperative treatment and proposed CRM of 2 mm as the adequate limit [8]. However, this study was criticized for the treatment heterogeneity of patients included for analysis despite large sample size. Considering the regression effect of the conventionally fractionated preoperative CRT [3], the prognostic significance or adequate length in the setting of preoperative CRT may be different from patients undergoing up-front surgery or short-course radiotherapy. Current study assessed the effect and adequate length of RM in a homogenous cohort of rectal cancer patients who underwent conventionally fractionated preoperative CRT and total meosrectal excision. In addition, present study only included patients with narrow margin (CRM ≤ 0.5 cm or DRM ≤ 1.0 cm). In this way, present study accrued more homogenous cohort without abundant and unnecessary data, because the prognosis of patients with CRM > 0.5 cm or DRM > 1.0 cm is reported to be steadily good and not related to the effect of RM [8,16,17].

All studied end-points were segregated with maximal difference at CRM of 1.5 mm in current study. The adequate length of CRM has been controversial between 1 mm and 2 mm [5,14,15]. In the similar patient group with long-course preoperative CRT, Trakarnsanga et al. recently reported that CRM of 1 mm is a cut-off value for local recurrence but 2 mm for distant recurrence [10]. While previous studies used simple comparison among arbitrarily divided groups, current study used continuous variable in micrometer dimension from microscopic measurements and analyzed RM with maximally selected rank statistics. As RM is a factually continuous variable and measurement in micrometer dimension is technically feasible, method used in current study could be considered as reasonable and statistically unbiased approach.

The second purpose of the present study was to assess the effect of treatment response on RM after preoperative CRT. In subgroup analysis based on the response to preoperative CRT, the impact of positive CRM was not significant in the good responders in contrast to the poor responders. As nearly all patients (94.0%) received postoperative chemotherapy as the institutional treatment protocol, different impact of CRM could be assessed as difference in tumor biology and/or effect of postoperative chemotherapy. In the good responders, residual tumor cells resulting in positive CRM might consist of responsive or impending non-viable tumor cells. This responsive biology of residual tumor cells may have lost the prognostic significance of CRM after postoperative chemotherapy. In contrast, the residual tumor cells at CRM for poor responders might be resistant or viable, and this could mean aggressive biology related to deteriorated prognosis and resistance to adjuvant chemotherapy. This finding suggests why despite positive CRM, survival of subgroup of patients, namely good responders, is comparable to that of patients with negative CRM.

While the treatment strategy compensating positive CRM has not been clearly established in previous studies [4,5], results of current study may lead to hypothesis that postoperative treatment may be individualized based on treatment response. Local treatment approach such as postoperative radiotherapy or CRT has failed to compensate for positive CRM according to previous studies [2,4]. Systemic therapy may be required because positive CRM is related with high risk for distant metastasis as demonstrated in the current study as well as other studies [6,7,9,10,14]. EORTC 22921 showed that the tumor biology could be linked to the effect of chemotherapy [18]. In this trial, patients downstaged by preoperative CRT were more likely to benefit from adjuvant chemotherapy. Results from current study, where nearly all patients (94.0%) received postoperative chemotherapy, also suggest that long-term survival may be expected for good responders, despite positive CRM. This may serve as a foundation for further studies to establish postoperative treatment strategy based on tumor biology for positive CRM.

Interestingly, DRM of 7 mm, which showed the maximal survival difference, was not prognostic for all studied end-points in both univariate and multivariate analysis. Previous pathologic studies reported that subclinical distal bowel intramural spreads are found within 1 cm distally from visible tumor [16,19]. Accordingly, 1 cm DRM has been recommended [15]. However, in the systemic review of Bujko et al., length of DRM was not correlated with recurrence rates or survival. So, it was concluded that <1 cm DRM did not jeopardize oncologic safety [17]. Particularly in the setting of preoperative treatment, other previous studies also proposed that <1 cm could be accepted without compromising clinical outcomes [20,21], and the result of present study in the setting of the conventionally fractionated preoperative CRT also supports this notion. Thus, narrow DRM defined as <1 cm could be acceptable for the patients undergoing the conventionally fractionated preoperative CRT.

Present study is not free from limitations. First, although all patients were treated with similar protocol at single institution, not all patients underwent postoperative chemotherapy as described above. Secondly, although the distribution of subgroup was well balanced for significant prognostic factors, due to retrospective nature of the study design, some of possible statistical bias may have not been removed. Thirdly, present study lacked comparative group without adjuvant treatment to confirm the role of adjuvant chemotherapy for patients with positive CRM. Therefore, suggested influence of the treatment response on the impact of CRM could be a promising hypothesis for further studies with larger cohort, but this needs to be validated.

## Conclusions

Close CRM, defined as 1.5 mm, was a significant prognosticator, but the impact was different for treatment response to preoperative CRT. Postoperative treatment strategy may be individualized based on this finding. However, findings from this study need to be validated with larger cohort.

## Competing interests

The authors declare that they have no competing interests.

## Authors’ contributions

EKC contributed to conception and design of the study, and revised the manuscript. JHL, KK and SWH contributed to analysis and interpretation of data, and drafted the manuscript. SJ, KJP, JP, GHK, SH, DO, SI, TK, and YB participated in data acquisition and literature research. All authors read and approved the final manuscript.

## Pre-publication history

The pre-publication history for this paper can be accessed here:

http://www.biomedcentral.com/1471-2407/13/576/prepub
